# High Rates of Hidden HCV Infections among Hospitalized Patients Aged 55–85

**DOI:** 10.3390/pathogens10060695

**Published:** 2021-06-03

**Authors:** Annarita Valeria Piazzolla, Giulia Paroni, Francesca Bazzocchi, Mauro Cassese, Antonio Cisternino, Luigi Ciuffreda, Franco Gorgoglione, Leonardo Gorgoglione, Vincenzo Palazzo, Natale Sciannamè, Marco Taurchini, Pasquale Vaira, Giovanna Cocomazzi, Maria Maddalena Squillante, Filippo Aucella, Nicola Cascavilla, Salvatore De Cosmo, Michelantonio Fania, Antonio Greco, Antonio Laborante, Maurizio Leone, Evaristo Maiello, Mauro Salvatori, Lazzaro Di Mauro, Alessandra Mangia

**Affiliations:** 1 Liver Unit, IRCCS “Casa Sollievo della Sofferenza”, 71013 San Giovanni Rotondo, Italy; v.piazzolla@operapadrepio.it (A.V.P.); g.cocomazzi@operapadrepio.it (G.C.); m.squillante@operapadrepio.it (M.M.S.); 2 Blood Bank, IRCCS “Casa Sollievo della Sofferenza”, 71013 San Giovanni Rotondo, Italy; giulia.paroni@operapadrepio.it (G.P.); l.dimauro@operapadrepio.it (L.D.M.); 3 Abdominal Surgery, IRCCS “Casa Sollievo della Sofferenza”, 71013 San Giovanni Rotondo, Italy; f.bazzocchi@operapadrepio.it; 4 Cardiosurgery, IRCCS “Casa Sollievo della Sofferenza”, 71013 San Giovanni Rotondo, Italy; m.cassese@operapadrepio.it; 5 Urology, IRCCS “Casa Sollievo della Sofferenza”, 71013 San Giovanni Rotondo, Italy; antonio.cisternino@operapadrepio.it; 6 Breast Surgery, IRCCS “Casa Sollievo della Sofferenza”, 71013 San Giovanni Rotondo, Italy; l.ciuffreda@operapadrepio.it; 7 Orthopedics, IRCCS “Casa Sollievo della Sofferenza”, 71013 San Giovanni Rotondo, Italy; f.gorgoglione@operapadrepio.it; 8 Neurosurgery, IRCCS “Casa Sollievo della Sofferenza”, 71013 San Giovanni Rotondo, Italy; l.gorgoglione@operapadrepio.it; 9 Vascular Surgery, IRCCS “Casa Sollievo della Sofferenza”, 71013 San Giovanni Rotondo, Italy; v.palazzo@operapadrepio.it; 10 Gynaecology, IRCCS “Casa Sollievo della Sofferenza”, 71013 San Giovanni Rotondo, Italy; n.scianname@operapadrepio.it; 11 Thoracic Surgery, IRCCS “Casa Sollievo della Sofferenza”, 71013 San Giovanni Rotondo, Italy; m.taurchini@operapadrepio.it; 12 Intensive Care, IRCCS “Casa Sollievo della Sofferenza”, 71013 San Giovanni Rotondo, Italy; p.vaira@operapadrepio.it; 13 Nephrology, IRCCS “Casa Sollievo della Sofferenza”, 71013 San Giovanni Rotondo, Italy; f.aucella@operapadrepio.it; 14 Hematology, IRCCS “Casa Sollievo della Sofferenza”, 71013 San Giovanni Rotondo, Italy; n.cascavilla@operapadrepio.it; 15 Internal Medicine, IRCCS “Casa Sollievo della Sofferenza”, 71013 San Giovanni Rotondo, Italy; s.decosmo@operapadrepio.it; 16 Dermatology, IRCCS “Casa Sollievo della Sofferenza”, 71013 San Giovanni Rotondo, Italy; michele.fania@operapadrepio.it; 17 Geriatrics, IRCCS “Casa Sollievo della Sofferenza”, 71013 San Giovanni Rotondo, Italy; a.greco@operapadrepio.it; 18 Oftalmology, IRCCS “Casa Sollievo della Sofferenza”, 71013 San Giovanni Rotondo, Italy; a.laborante@operapadrepio.it; 19 Neurology, IRCCS “Casa Sollievo della Sofferenza”, 71013 San Giovanni Rotondo, Italy; m.leone@operapadrepio.it; 20 Oncology, IRCCS “Casa Sollievo della Sofferenza”, 71013 San Giovanni Rotondo, Italy; e.maiello@operapadrepio.it; 21 Cardiology, IRCCS “Casa Sollievo della Sofferenza”, 71013 San Giovanni Rotondo, Italy; m.salvatori@operapadrepio.it

**Keywords:** HCV, micro-elimination, screening, birth cohort, linkage-to-care, sofosbuvir/velpatasvir

## Abstract

Background and Aims: The WHO has solicited all countries to eliminate HCV by 2030. The Italian government started routine screening for HCV infection in January 2021, initially targeting subjects born between 1969 and 1989. With the aim of achieving micro-elimination, we designed a hospital-wide project focusing on inpatients born from 1935 to 1985 and conducted it in our institution. Method: All inpatients aged 35 to 85, admitted from 10 February 2020 to 9 February 2021 for many different diseases and conditions underwent HCV antibody (HCVAb) testing by third-generation ELISA. When positive, reflex HCV RNA testing and genotyping were performed. Clinical history, fibrosis diagnosis, laboratory data and concomitant medications were available for all. Results: The HCV screening rate of inpatients was 100%. In total, 11,748 participants were enrolled, of whom 53.50% were male. The HCVAb positivity rate was 3.03%. The HCVAb rate increased with age and was higher for patients born between 1935 and 1944 (4.81%). The rate of HCV RNA positivity was 0.97%. The vast majority (80.70%) of HCV RNA-positive participants were 55 or older; in about 40% of cases, HCV RNA-positive patients were unaware of their infection. Although 16 patients died after HCV chronic infection diagnosis (two due COVID-19) or HCV treatment prescription (one due to COVID-19), 74.56% of patient HCV diagnoses were linked to HCV treatment, despite their co-morbidities. All patients older than 65 who died had an active HCV infection. Conclusion: The present study revealed a rate of active HCV infections among inpatients lower than what has been reported in the past in the general population; this appears to be a result of the widespread use of pangenotypic direct-acting antiviral agents (DAAs). The overall rate of active infection was lower than the rate observed in the 1935–1954 cohort. The high rate of inpatients unaware of HCV infections and the high number of deaths among subjects with an active HCV infection born from 1935 to 1954, suggest that, at least in southern Italy, targeted screening of this birth cohort may be required to reduce the number of undiagnosed cases and hidden infections.

## 1. Introduction

Hepatitis C virus (HCV) affects more than 71 million people worldwide, representing a serious public health issue. Based on the efficacy of new DAAs, ensuring viral clearance in more than 95% of treated patients, the WHO has promoted the Global Sectorial Strategy for HCV elimination by 2030, with the ambitious objective of increasing diagnosis to 90% and reducing HCV-related deaths by 65% [1]. Chronic HCV infection can remain asymptomatic until the latest stages, leading to poor awareness of patient’s infection status and to a significant proportion of undiagnosed diseases. In Europe, 60% of all HCV-infected patients remain undiagnosed, with considerable differences across countries [2]. Key aspects preventing HCV elimination are the lack of reliable and updated estimates of active HCV infections and related disease burdens across Europe [3]. In Italy, the most recent HCV epidemiological studies date from before the use of DAAs became widespread in 2017 and showed a 1.7% prevalence; there was significant variability between the north and south and between cities and peripherical areas [4]. It was also reported that 87% of patients who were unaware of having HCVAbs were from the south [4].

Micro-elimination strategies targeting small fractions of the population, as opposed to nation-wide elimination programs, demonstrated that improved diagnosis and treatment rates resulted in great benefits in scaling up the HCV care cascade and in achieving WHO objectives. The identification of patients unaware of their HCV status represents the primary barrier that needs to be overcome [5]. In Italy, based on different HCV prevalence rates across geographical regions, the ideal screening strategy to identify undiagnosed HCV patients is an object of debate [5]. Until recently, guidelines have recommended the screening of high-risk patients; however, the new national screening campaign focuses on people born between 1989 and 1969 [6,7]. The epidemiology of HCV infections in Italy is linked to transmission routes and differs between geographical areas. In the north, young people with a history of substance use disorders are the main reservoir of undiagnosed infections, whereas, in the south, undiagnosed and inapparent infections peak among older persons, acquired either through intrafamilial or parenteral transmission [8]. Consequently, the most effective strategy to identify undiagnosed patients with chronic HCV infection is disputed; community-wide screening strategies have been explored at local levels [9], which have recently been linked to COVID-19 screening in the contemporary coronavirus pandemic [10].

Hospitalized patients are at high risk from hepatitis C and may represent the ideal population for cost-effective HCV screening and care in the south. In Puglia, the current real prevalence of HCV infection in the general population remains undetermined, but it is expected to be too low to justify community-wide screening [4,11]. The HCV-Free Hospital project has sought to improve testing and treatment interventions for patients at their first hospitalization. Our aim was to improve the HCV care cascade and to shed light on the prevalence of HCV infection in unselected patients hospitalized over a 12-month period in the south of Italy.

## 2. Patients and Methods

### 2.1. Study Design

Inpatients aged 35–85, consecutively admitted to different units of our institution, were offered HCVAb screening.

The study started on February 2020, just before the outbreak of the COVID-19 pandemic in Italy and might offer an example on how to reduce the negative impacts of the COVID-19 pandemic on HCV screening, although the COVID-19-positive case rate in our province was 6000/100,000 inhabitants [11], much lower than that of 10,000/100,000 inhabitants reported in northern Italy. An awareness campaign was launched within the hospital before the launch of this program and media advertisements and interviews were promoted at regional level.

Recruitment was carried out in the hospital after approval from the local Ethical Committee. Patients older than 85 and younger than 35 were excluded due to epidemiological and cost-effectiveness considerations [12,13,14,15]. Patients referred to the Gastroenterology and Hepatology units were also excluded. Patients who had already been hospitalized with HCV infection in the past were excluded. Overall, 12,261 participants were recruited; 513 patients were excluded because they did not meet the inclusion criteria. Due to the COVID-19 pandemic, this number was lower than the 19,000 expected on the basis of admissions in the previous year; however, the only amendment adopted concerned televisit monitoring, for patients not allowed to reach the hospital during lockdown. All the participants consented to be tested. Clinical history, diagnosis, laboratory data and concomitant medications were available for all. In all patients, treatment was performed using sofosbuvir/velpatasvir (SOF/VEL) with or without ribavirin for 12 weeks. According to our strategy and in favor of a simplified approach, no treatment monitoring was performed in patients with compensated liver disease eligible for treatment. Monthly follow-up calls were performed during the pandemic. Adhering to local administrative rules, the home delivery of drugs for all chronic treatment types was adopted during the pandemic; therefore, no protocol amendments were required. Patients with decompensated liver diseases, or oncologic diagnosis taking co-medication, were seen every 4 weeks, with the only exception being those who could not reach the hospital during the COVID-19 pandemic and were followed using telehealth.

### 2.2. Type of Testing

Third-generation ELISA HCVAb assays (ORTHO Diagnostics, Raritan, NJ, USA) were performed used in all consecutive patients admitted to the hospital. In cases of borderline values, a confirmatory RIBA test (INNOLIA HCV) was performed. Patients testing positive underwent reflex HCV RNA quantitative testing using the Abbott assay, with a lower limit of detection of 12 IU/mL. Genotyping was also performed by INNOLIPA HCV (Innogenetics, Gent, Belgium).

### 2.3. Study Outcomes

Primary outcome measures included the performed hepatitis C antibody tests and HCV RNA assessment. Patients with positive HCV RNA results and a reactive antibody test were subjected to genotype assessment and were offered treatment with a pangenotypic single-pill combination of SOF/VEL for 12 weeks, with or without ribavirin according to the severity of the liver damage. Sustained virological responses 12 months after the end of treatment (SVR12) and at the post-treatment follow-up were considered secondary outcomes.

### 2.4. Statistical Analysis 

Prevalence rates and their 95% confidence intervals (CIs) were calculated. Qualitative or quantitative variables were analyzed using non-parametric tests such as the chi-squared test, Kruskal–Wallis test, or the Mann–Whitney test, where appropriate. Using logistic regression, odds ratios (ORs) together with the corresponding 95% CIs were computed for the majority of the investigated variables. All *p* values were based on two-tailed tests and *p* < 0.05 was considered to be statistically significant. Statistical analysis was performed using SPSS version 23 software (SPSS Inc, Chicago, IL, USA).

## 3. Results

**Figure 1:**
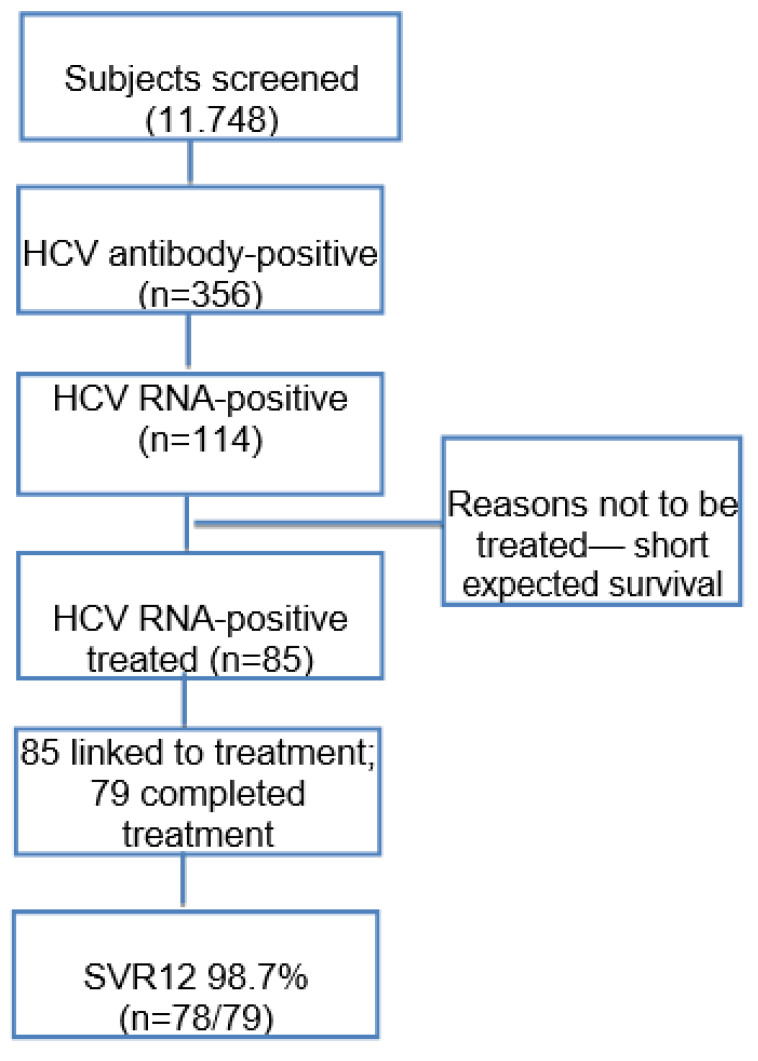
HCV care cascade for the 11,748 subjects involved in this study.

**Figure 2:**
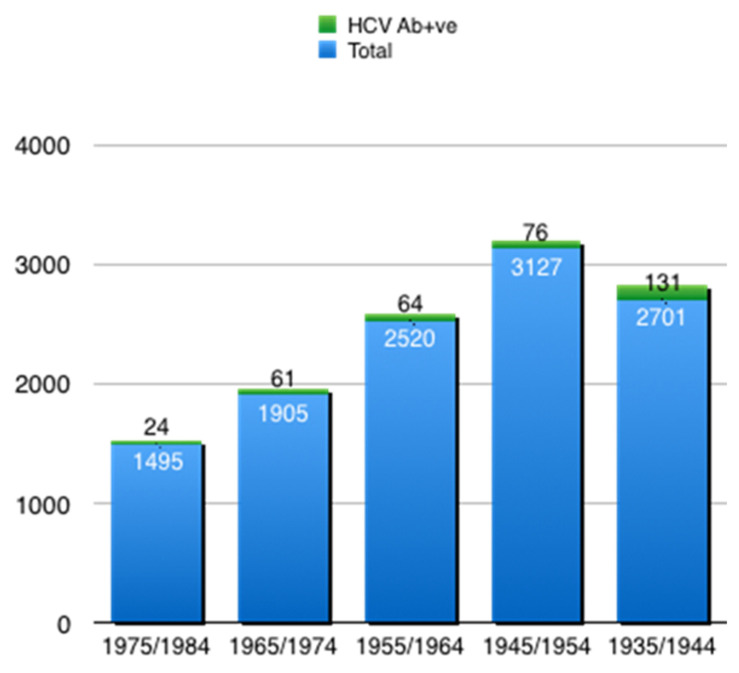
HCVAb-positive subjects by birth cohorts.

The study population included 11,748 subjects (Figure 1), of whom the majority were male (53.50%). The mean age was 64.6 ± 12.1 years. The number of patients admitted was partially affected by the COVID-19 pandemic: there was a 36.10% reduction in the total number of patients admitted to the hospital, across many different diseases and conditions, in comparison to the previous year. Overall, 356 (3.03%) had a positive HCVAb result (Table 1). The rate of HCVAb-positive males was significantly higher than that of females (60.41% vs. 39.60%, *p* = 0.047). The number of positive HCVAb results with respect to age cohorts is reported in Figure 2. The positivity rate increased with age, confirming the already-known birth cohort effect associated with HCV infection in Italy [13,15]. For subjects born from 1935 to 1944, the 4.8% HCVAb positivity rate was significantly higher than for subjects born 10 or 20 years earlier (*p* = 0.0001), with prevalence rates of 2.4% and 2.5%, respectively. An increment of 3.20% in the rate of HCVAb positivity was observed among subjects of birth cohort 1965–1974. This was lower than the rate observed among the oldest cohort (*p* = 0.006), but not different from the peaks observed among people born between 1945 and 1954 or 1955 and 1964. Overall, 271 of 356 (76.1%) HCVAb positive patients were 56 or older (Table 2). 

Of the 11,748 subjects tested, 114 were HCV RNA-positive, as shown in Figure 1. This rate represents 0.97% of the overall population and 32.02% of HCVAb-positive subjects (Table 1). Again, the highest rate was observed in patients born between 1935 and 1944. The lowest rate, 0.40%, was registered among people born from 1975 to 1985 (Table 2), who are currently, the object of the national screening campaign. 

Of the 356 HCVAb-positive patients, 167 (46.91%) were unaware of their HCV status. Of the other 189 HCVAb-positive patients aware of their HCV status, 104 had been treated, 6 of whom without success.

Among the 114 HCV RNA-positive patients, 44 (38.59%) were unaware of their HCV status, suggesting that a high proportion of active HCV infections in the south of Italy remain undiagnosed. Overall, 70 out of 114 patients with an active infection were aware of their condition, but had chosen not to start treatment (47.10%) or had forgotten about their condition. As shown in Table 1, the most prevalent genotype was genotype 1 (GT1) (52.63%), followed by GT2 and GT3. Among HCVAb-positive participants, 15.44% of patients displayed evidence of advanced liver disease; this rate increased to almost 22.80% among patients with an active HCV infection, 38.46% of whom had decompensated cirrhosis. Overall, 23 HCVAb-positive patients died during hospitalization or within 42 days from diagnosis; in only 2 cases, the death was related to COVID-19. Of them, 20 were older than 65. All 16 HCV RNA-positive subjects who died were older than 65.

### 3.1. HCVAb Positivity by Admission Unit

The distribution of HCVAb- and HCV RNA-positive patients by admission unit is reported in Table 3. The highest frequency of HCV RNA-positive results was observed among patients admitted to the Orthopedics Unit for trauma events (14.91%). The rate of HCV RNA-positive participants linked to care varied by admission unit and depended on co-morbidities. The strongest correlation with care rate (92.30%) was registered among subjects admitted to the Urology Unit. The proportions of HCV RNA-positive patients were equal in the surgical and medical departments; however, the rate of unaware patients with an active HCV infection among subjects admitted to the Orthopedics or Urology units was higher than for those admitted to other medical units, suggesting that although the former units may better reflect the epidemiology of the general population southern Italian with HCV infection, unawareness represents the main barrier for treatment. For patients admitted to the Oncology Unit—which had the highest rate of unaware patients among all medical units—the number of these cases linked to care was the lowest, with 6 out of 12 candidates dying before the start/completion of treatment. In the majority of these cases, death was due to the progression of the disease which had caused hospitalization. In three patients admitted to the Internal Medicine Unit, treatment had to be delayed by 15–30 days due to COVID-19 infection.

### 3.2. HCV RNA Positivity by Region of Origin

HCV RNA positivity was analyzed by the region of origin of the patients evaluated. Given the large catchment area of our hospital, we had the opportunity to analyze the distribution of undiagnosed cases by region of origin. The highest numbers of extra-regional patients were from Calabria, followed by Molise, Basilicata and Abruzzo. Of the 250 patients from Calabria, 1.21% had positive HCVAb results and were treated. Similar rates were observed for patients from Basilicata and Molise, with rates of active HCV infection of 1.52% and 1.39%, respectively.

### 3.3. Patients Whose Infections Were Not Linked to Treatment

Of the 114 patients with active HCV infections, 85 (74.56%) were linked to care (Figure 1), but only 82 began treatment (one refused and two died due to the progression of chronic kidney disease (CKD) and liver cancer before starting treatment). Overall, 29 of the 114 patients’ infections (25.43%) were not linked to treatment (Table 4). Out of these 29, 16 patients died before any potential therapy could be prescribed, in addition, to those who did not start treatment. Deaths in this group were mainly related to cancer, cardiovascular or kidney diseases: one patient with a history of alcohol and IV substance use died of hepatocellular carcinoma (HCC); another patient with cirrhosis, Child–Pugh class B, died of sepsis. Underlying diseases could potentially explain why an additional 13 who were not treated are reported in Table 4. All but one patient in this group was male and the vast majority were infected with GT1 and unaware of their HCV condition.

### 3.4. Televisit Monitoring

A monthly telehealth follow-up was utilized to monitor 13 (15.85%) of the 82 patients whose infections were linked to care and had previous diagnoses of cancer (*n* = 7), severe cardiovascular diseases (*n* = 4), or advanced decompensated cirrhosis (*n* = 2); these patients could not attend our clinic during the treatment period due to the COVID-19 pandemic. Laboratory evaluations were performed at the patients’ houses with the assistance of dedicated nurses and test results were sent by mail to our unit. Patients were contacted by video call every 4 weeks. Treatment was completed in all patients apart from one, who died of sepsis due to decompensated liver disease. One patient with cirrhosis and a history of intravenously injecting narcotic substances discontinued treatment. 

### 3.5. SVR12 in Patients Able to Complete the Assigned Treatment

Of the 85 candidates who were eligible to start treatment, 3 did not (due to personal reasons in a previous intravenous drug-user (IVDU) and due to the worsening of their underlying diseases in the other two). After the treatment began, one patient died at week 8 due to COVID-19 infection and another died of lung cancer several days before the end of HCV treatment. The effectiveness of the SOF/VEL pangenotypic combination was evaluated in our study in a total of 79 of the 85 infections linked to care; one more patient died of sepsis due to decompensated liver disease shortly before week 4 of follow-up. As shown in Figure 1, of the 79 who completed 4 weeks of follow-up, 78 achieved SVR12 (98.73%). The only non-responder was a patient with lung cancer who was 70% adherent. All other patients, despite seven of them requiring concomitant chemotherapy for severe underlying neoplastic diseases, were able to achieve SVR12. No drug–drug interactions were observed.

## 4. Discussion

Our HCV-Free Hospital program identified patients whose infections would have been missed, including some who were particularly frail. Despite co-morbidities causing hospitalization, 74.56% of these patients’ infections were linked to care and were successfully cured upon the completion of treatment. Part of the study success was linked to the opportunity to monitor patients using telehealth during the COVID-19 pandemic. 

The 0.97% rate of active HCV infection among hospitalized patients observed in this study, was lower than the 1.7% prevalence previously reported in the general Italian population in [4], although it is in agreement with the 0.8% reported in the latest European epidemiological reports [16]. In our study, a significant association between age and HCV active infection was observed, confirming the already-known birth cohort effect for HCV infection in Italy [13]. In particular, in patients older than 55 years, the number of active infections in our study mirrored the number of patients dying due to active infection. Considering that patients aged between 55 and 65 are people not yet retired, they generally have active social lives with a potential to transmit HCV infection; thus, our results suggest that it would be advisable to extend the age range of the subjects currently, the focus of the national HCV screening campaign, despite recent models suggesting that the cost benefit in older patients might not be as evident as in younger patients [6]. Moreover, given the high rate of chronic disease reported in Italy [17], curing HCV infection before the occurrence of severe co-morbidities such as diabetes or cardiovascular diseases, accounting for 37.5% of the failures linked to care in this study, may be very relevant. Finding HCV-infected patients with co-morbidities as early as possible, in addition, to reducing the number of HCV cases [18], may benefit the treatment of newly occurring concomitant diseases or complications of pre-existing conditions. The cohort effect was largely demonstrated in the population of the south of Italy as compared to those in the north; reflections of incorrect practices in the past were further confirmed in this study, whereas improved social behavior could explain the lower HCV rate registered in the younger population [19,20]. 

Various initiatives focusing on birth cohort screening have demonstrated the cost-effectiveness of this approach [20]. As demonstrated in a review by Morgan et al., examining eight studies, this approach was shown to be more effective than risk-based testing [21]. Hospital screening has been pursued in various countries, including the United States. Other diverse approaches have been utilized, including screening limited to the Emergency Units [21,22,23]. In the United State, during the COVID-19 pandemic, automated viral hepatitis screening significantly scaled up linkages to care and treatment, proving that no additional resources from the hospital staff needed to be diverted [22]. Our study confirmed that these programs did not need to be halted during the pandemic and were strategically managed with the help of telemedicine. 

In an ongoing study in Spain, at the emergency department of a tertiary healthcare center in Barcelona, the prevalence of HCV RNA-positive subjects was not higher than 0.67%, although this is twice as high as the overall 0.22% rate reported for the Spanish population [24]. Both the proportion of patients unaware of their infection and the advanced age of patients first discovered to be infected with HCV were similar to those reported in our study.

In a study conducted two years before ours, in 2017–2018 at Venezia-Mestre Hospital in northern Italy, a prevalence rate of 2.1%, which was 2–3 times higher than that in the general population at the time, was reported. Unfortunately, data on HCV RNA results in that study were available for only 23% of patients, preventing any comparisons from being made [25]. The dramatic impact of HCV pangenotypic treatment launched in 2017 has increased the number of HCV-infected Italians treated [26] and explains the lower rate registered today. In contrast, to previous models, our study showed that despite the particularly vulnerable population identified, HCV eradication might be cost-effective if more than merely a money-saving strategy is applied. These patients need HCV treatment not only to reduce the risk of liver disease complications, but also to allow the completion of their concurrent oncologic treatments or to prevent the inapparent transmission of HCV infection during repeated hospitalizations [27]. In contrast, to COVID-19 infection, which had a limited impact on the mortality of our patients, active HCV infections seem to represent an additional risk factor in people of advanced age with cardiovascular, kidney or oncological comorbidities. 

The other important finding in our study concerns awareness. Of the HCV-infected patients identified, those unaware of their HCV status represented a high proportion, nearly 40%. In older patients, lower education levels and limited access to media and the internet could explain undiagnosed diseases [28], whereas the fear of side effects might explain patients missing second courses of treatment because of a lack of success from using IFN-based regimens in the past [29]. Consistent with other evidence [30], our study suggests that attempts to increase diagnoses continue to be worthwhile, given the low rate of diagnosis recently shown by the European Center for Disease Control [31]. In Italy, although models projecting epidemiological data derived from cities might suggest young populations to be a target for screening campaigns [31], evidence gathered in rural areas or in different geographical regions suggest that the optimal targets may differ. Despite our successful initiative, the number of patients treated in this program was limited due to the high mortality and morbidity rates of newly diagnosed patients, including the particularly vulnerable population admitted for cancer, for cardiovascular diseases, or for severely advanced liver diseases. This experience may represent the first step towards age-based, cost-effective screening, delivering access to HCV treatment and connections to care before hospitalization due to co-morbidities.

This study has some weaknesses and strengths. The data obtained were from a single center and our findings may be different from those in other institutions in northern Italy. Based on our combined expertise, we felt confident in prescribing HCV treatments based on consultations during the hospital stay and without additional in-person visits. In keeping with other reports [32], the telehealth approach was vital during the COVID-19 pandemic; frail patients, such as those with severe cardiovascular disease and decompensated cirrhosis, could continue to receive treatment without having to attend the hospital [33]. The devolution of HCV care after targeted screening to specialized hepatology centers which adopt telemedicine may beneficial for reaching and managing vulnerable populations with serious co-morbidities and taking a large number of concomitant medications. This model could be adopted in other hospitals aiming for the micro-elimination of HCV and would ensure an exponential effect based on the increased awareness.

In conclusion, our hospital-centered approach for HCV micro-elimination offers a framework for scaling-up linkages to care and treatment on the path towards the achievement of WHO goals by 2030. The prevalence of active HCV infection in our program was lower than rates in the past but still high and our results suggest that HCV screening should be extended in Italy to patients aged from 55 to 75. This strategy would be cost-effective as compared to universal screening. Frail populations require dedicated screening and integrated care programs, which can start from hospitals and continue in a network involving telehealth processes.

## Figures and Tables

**Table 1:** Baseline characteristics of the 356 HCVAb-positive patients and 114 HCV RNA-positive patients.

Characteristics	HCVAb-Positive (N = 356)	HCV RNA-Positive (N = 114)
Age, years, mean (SD)	68.2 (12.9)	70.5 (13.0)
Sex, male, *n* (%)	215 (60.4) *	60 (52.6)
female, *n* (%)	141 (39.6)	54 (47.3)
Cirrhosis, *n* (%)		
F4	55 (15.4) **	26 (22.8)
F1–F2–F3	301 (84.6)	88 (77.2)
HCV, *n* (%)		
GT 1	not applicable	60 (52.6)
GT 2		40 (35.1)
GT 3		10 (8.7)
GT 4–6		4 (3.6)
IV drug use, former or current, *n* (%)		
Yes	31 (8.7)	16 (14)
No	325 (91.2)	98 (86)
Time from HCV RNA diagnosis to SOF/VEL treatment, median days (range)	not applicable	62 (25–154)

* *p* = 0.047; ** *p* = 0.015.

**Table 2:** Prevalence of HCVAb positivity by birth decades and deaths by HCV RNA status.

Age Cohort	Number of Subjects	HCVAbs Positivity *n*, (%)	HCV RNA Positivity *n*, (%)	Deaths among HCVAb-Positive Patients *n*, (%)	Deaths among HCV RNA-Positive Patients *n*, (%)
1975–1984	1495	24 (1.6)	6 (0.4)	1 (4.2)	0
1965–1974	1905	61 (3.2)	16 (0.8)	1 (1.6)	0
1955–1964	2520	64 (2.5)	15 (0.6)	1 (1.6)	0
1945–1954	3127	76 (2.4)	23 (0.7)	5 (6.6)	5 (21.7)
1935–1944	2701	131 (4.8)	54 (2.0)	15 (11.4)	11 (20.4)

**Table 3:** HCV RNA positivity by admission units and awareness.

Characteristics	HCVAb-Positive Total (N = 356)	HCV RNA-Positive Total (N = 114)	HCV RNA-Positive Unaware (N = 44)
Surgical Units, *n* (%)	182 (51.1)	59 (51.8)	24 (54.5)
Urology	32 (9.0)	13 (11.4)	8 (18.1)
Orthopedics	28 (7.9)	17 (14.9)	9 (20.4)
Thoracic surgery	14 (3.9)	5 (4.3)	2 (4.5)
Cardiosurgery	16 (4.5)	3 (2.6)	1 (2.3)
Ophthalmology	3 (0.8)	1 (0.8)	0
Breast surgery	3 (0.8)	3 (2.6)	1 (2.3)
Gynecology	18 (5.1)	4 (3.5)	1 (2.3)
General surgery	43 (12.1)	8 (7.0)	0
Maxillofacial	15 (4.2)	2 (1.7)	1 (2.3)
Neurosurgery	10 (2.8)	3 (2.6)	1 (2.3)
Medical Units, *n* (%)	174 (48.9)	55 (48.2)	20 (45.5)
Hematology	14 (3.9)	4 (3.5)	2 (4.5)
Oncology	20 (5.6)	12 (10.5)	7 (13.6)
Nephrology/Dialysis	19 (5.3)	5 (4.3)	0
Cardiology	20 (5.6)	7 (6.1)	3 (6.8)
Internal Medicine	16 (4.5)	10 (8.7)	3 (6.8)
Gerontology	3 (0.8)	3 (2.6)	1 (2.3)
COVID-19 Unit	27 (7.6)	8 (7.1)	2 (4.5)
Dermatology	23 (6.5)	3 (2.6)	1 (2.3)
Neurology	1 (0.3)	1 (0.8)	1 (2.3)
Intensive care	6 (1.7)	0	0
Thalassemia unit	12 (3.4)	0	0
Occupational medicine	13 (3.7)	2 (1.7)	0

**Table 4:** Baseline characteristics of patients who were not linked to treatment.

Characteristics	Patients’ Infections Not Linked to Care (N = 29)
Sex, male, *n* (%)	27 (93.1)
Fibrosis stage, *n* (%)	
F0–F2–F3	21 (72.4)
F4	8 (27.5)
Treatment history, *n* (%)	
Unaware	26 (89.6)
Treatment-experienced (DAA-naïve)	3 (10.4)
HCV, *n* (%)	
GT 1	26 (89.6)
GT 2	1 (3.4)
GT 3	2 (6.8)
Underlying conditions	
substance abusers (former or current), cirrhosis/HCC	
*n* (%)	
Not starting treatment ^	7 (24.1)
Oncologic diseases, *n* (%) *	
Not starting treatment	9 (31.0)
Cardiovascular diseases/diabetes, *n* (%) ^§^	
Not starting treatment	10 (34.4)
Chronic kidney diseases, *n* (%) °	
Not starting treatment	3 (10.3)

^§^ 8/10 died, one of them had COVID-19 infection; ° 1/3 died; * 5/9 died, one of them had COVID-19 infection; ^ 2/7 died, one each of HCC and Child–Pugh class B cirrhosis, respectively.

## Data Availability

Data are available at the institutional repository site.
